# Analysis of Cyclic Imines in Mussels (*Mytilus galloprovincialis*) from Galicia (NW Spain) by LC-MS/MS

**DOI:** 10.3390/ijerph17010281

**Published:** 2019-12-31

**Authors:** Guillermo Moreiras, José Manuel Leão, Ana Gago-Martínez

**Affiliations:** 1 Department of Analytical and Food Chemistry, Faculty of Chemistry, Campus Universitario de Vigo, University of Vigo, 36310 Vigo, Spain; gmoreiras@uvigo.es (G.M.); leao@uvigo.es (J.M.L.); 2 European Union Reference Laboratory for Marine Biotoxins (EURLMB), CITEXVI, Campus Universitario de Vigo, 36310 Vigo, Spain

**Keywords:** cyclic imines (CIs), spirolides (SPXs), pinnatoxins (PnTXs), liquid chromatography-tandem mass spectrometry (LC-MS/MS), solid phase extraction (SPE), shellfish

## Abstract

Cyclic imines (CIs) are being considered as emerging toxins in the European Union, and a scientific opinion has been published by the European Food Safety Authority (EFSA) in which an assessment of the risks to human health related to their consumption has been carried out. Recommendations on the EFSA opinion include the search for data occurrence of CIs in shellfish and using confirmatory methods by liquid chromatography-tandem mass spectrometry (LC-MS/MS), which need to be developed and optimized. The aim of this work is the application of LC-MS/MS to the analysis of gymnodimines (GYMs), spirolides (SPXs), pinnatoxins (PnTXs), and pteriatoxins (PtTXs) in mussels from Galician Rias, northwest Spain, the main production area in Europe, and therefore a representative emplacement for their evaluation. Conditions were adjusted using commercially available certified reference standards of GYM-A, SPX-1, and PnTX-G and evaluated through quality control studies. The EU-Harmonised Standard Operating Procedure for determination of lipophilic marine biotoxins in molluscs by LC-MS/MS was followed, and the results obtained from the analysis of eighteen samples from three different locations that showed the presence of PnTXs and SPXs are presented and discussed. Concentrations of PnTX-G and SPX-1 ranged from 1.8 to 3.1 µg/kg and 1.2 to 6.9 µg/kg, respectively, and PnTX-A was detected in the group of samples with higher levels of PnTX-G after a solid phase extraction (SPE) step used for the concentration of extracts.

## 1. Introduction

**Figure 1:**
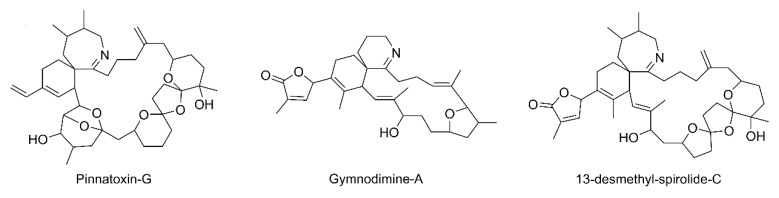
Structures of representative cyclic imines with commercially available standard solutions used for liquid chromatography-tandem mass spectrometry (LC-MS/MS) analysis.

Gymnodimines (GYMs), spirolides (SPXs), pinnatoxins (PnTXs), pteriatoxins (PtTXs), prorocentrolides, spiro-prorocentrimine, portimines, and symbioimines belong to cyclic imines (CIs), a family of marine biotoxins produced by dinoflagellates and accumulated in shellfish [1,2,3]. These compounds share as a common structural motif an imine group in a cyclic moiety, which has been identified as a pharmacophore with biological activity [4,5]. CIs have a mode of action based on the inhibition of nicotinic acetylcholine receptors, and although neurotoxic effects were observed in toxicological assays, as of yet there has been no reported information about human intoxication linked to their assimilation [6,7,8]. CIs are distributed worldwide in diverse geographical regions, including European waters, where compounds that belong to the GYM, SPX, and PnTX groups have been detected in shellfish from several countries [9,10,11,12,13,14,15]. In Spain, the third largest mussel producer of the world in 2017 [16], PnTX-G and 13-desmethylSPX-C (SPX-1) were found in samples from Catalonia (northeast Spain, Mediterranean Sea) [17] while in Galicia (northwest Spain, Atlantic Ocean), the main production area in the country, SPX-1 was detected [18] and PnTXs have been recently reported [19,20]. The European Food Safety Authority (EFSA) has developed a series of scientific opinions related to marine biotoxins, both for regulated and for emerging groups. The EFSA opinion for CIs includes several recommendations regarding the development and optimization of LC-MS/MS methods for their confirmation and for generating more information on the occurrence of these compounds in shellfish [21]. LC-MS/MS recently became the reference method for the control of lipophilic toxins in the European Union. The availability of certified reference materials for CIs as GYM-A, SPX-1, and PnTX-G (Figure 1) has allowed them to be included with other groups of marine lipophilic toxins that are currently legislated for the purpose of presently available reference methods [22,23,24,25]. In addition, because CIs include a long list of compounds and most of them are without commercially available standard solutions, LC-MS/MS methods based on fragmentation pathways of reference toxins have been proposed in order to study their occurrence [17,26]. This work was focused on the application of LC-MS/MS for the analysis of GYMs, SPXs, PnTXs, and PtTXs in mussels (*Mytilus galloprovincialis*) from Galician Rias in order to allow for the confirmation of PnTX-G and SPX-1 using standard solutions, as well as the detection of PnTX-A based on fragmentation pathways and key *m*/*z* ions.

## 2. Materials and Methods

### 2.1. Reagents and Standards

Certified reference standard solutions of gymnodimine A (2.50 ± 0.13 µg/mL) and pinnatoxin G (1.92 ± 0.09 µg/mL) were acquired from National Research Council (NRC) (Halifax, NS, Canada) and 13-desmethyl spirolide C (7.23 ± 0.10 µg/mL) was purchased from CIFGA S.A. (Lugo, Spain).

The acetonitrile and methanol obtained from Merck (Madrid, Spain) and the water obtained from J.T. Baker-Avantor (Madrid, Spain) were LC-MS grade. Formic acid (98–100% purity) and ammonium formate (≥99% purity) were acquired from Sigma-Aldrich (Madrid, Spain). The methanol and water used for sample treatment were analytical grade from Fisher Scientific (Madrid, Spain).

### 2.2. Sampling

**Figure 2:**
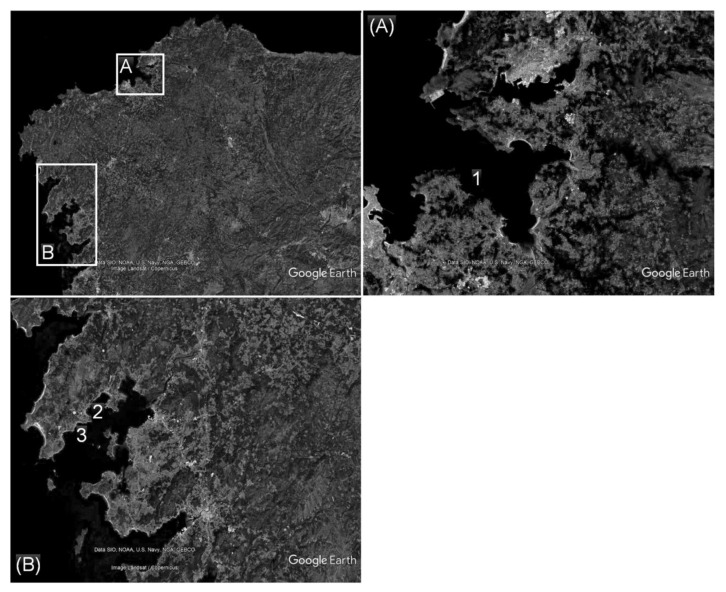
Map of Galicia (northwest Spain) with the sampling locations (1, 2, and 3) in Ría de Sada (**A**) and Ría de Arousa (**B**). Images (data SIO, NOAA, U.S. Navy, NGA, GEBCO; image Landsat/Copernicus) are from Google Earth.

Mussels (*Mytilus galloprovincialis*) were collected from Ría de Sada and Ría de Arousa (Figure 2) during May 2015. Fresh samples were carried to the laboratory under refrigeration (5 ± 3 °C) and were kept at −20 °C before analysis.

### 2.3. Extraction

Extraction was performed following EU-Harmonised Standard Operating Procedure (SOP) for determination of lipophilic marine biotoxins in molluscs by LC-MS/MS [27]. The sample extracts were filtered through a 0.22-µm filter before being analyzed in the LC-MS/MS equipment for the quantification of CIs.

### 2.4. Hydrolysis

In order to detect and quantify the esterified forms of PnTXs, an alkaline hydrolysis of sample extracts was performed according to the European Union Reference Laboratory for Marine Biotoxins (EURLMB) SOP [27] and filtered through a 0.22-µm filter before LC-MS/MS analysis.

### 2.5. Solid Phase Extraction (SPE) Step for the Concentration of CIs and Clean-Up of Sample Extracts

An aliquot of 10 mL of extract was dried under N_2_ stream at 40 °C. After dissolving in 4 mL of MeOH-H_2_O at 25%, the extract was loaded in a Strata-X cartridge, 30 mg/1mL (Phenomenex, Torrance, CA, USA) previously conditioned with 1 mL of MeOH and 1 mL of H_2_O. The wash step consisted of the addition of 1 mL of MeOH-H_2_O at 50%, following which the stationary phase was dried for 30 s. After that, elution was performed with 2 × 0.5 mL of MeOH with 2% of formic acid and filtered through a 0.22-µm filter for LC-MS/MS analysis.

### 2.6. LC-MS/MS Analysis

Chromatographic separation was applied on an Agilent 1290 Infinity (Agilent Technologies, Waghaeusel-Wiesental, Germany) using an Agilent Zorbax SB-C8 Rapid Resolution HD (2.1 × 50 mm, 1.8 µm) at 40 °C. Mobile phase A was 100% water and B was 95% acetonitrile/water, both containing 2 mM ammonium formate and 50 mM formic acid. A gradient elution program with a flow rate of 0.4 mL/min was run starting with 20% B moving to 50% B in 5 min, and then a linear gradient was applied to return to the initial conditions. The total run time was 6 min, including an equilibration time of 1 min before the next injection. The samples and standard solutions in the autosampler were cooled to 4 °C, and a volume of 5 μL was injected.

MS detection was performed using an Agilent G6460A triple quadrupole mass spectrometer equipped with a Jet Stream ESI source. The capillary voltage and nozzle were set to 5 kV and 0 kV, respectively. A drying gas flow of 6 L/min at 200 °C, a nebulizer gas pressure of 50 psi (Nitrogen Generator System, Zefiro 40, Evry, France), and a sheath gas (nitrogen 99.999% pure, Airliquide, Porriño, Spain) flow of 12 L/min at 400 °C were used. The MS/MS and selected reaction monitoring (SRM) data acquisition conditions are shown in Table 1.

## 3. Results and Discussion

### 3.1. Adjustment of LC-MS/MS to the Analysis of CIs

**Figure 3:**
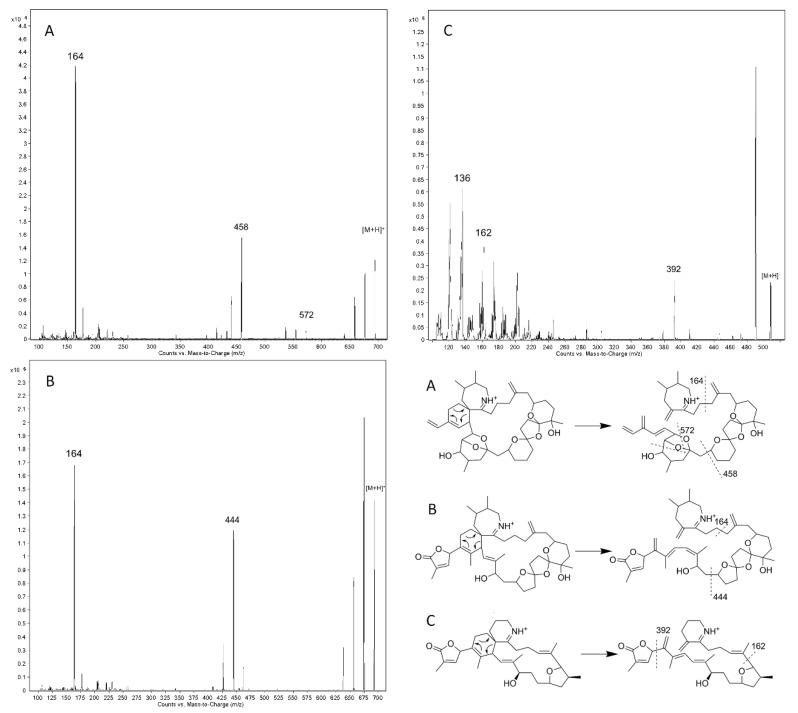
Product ion spectra obtained with a collision energy of 40 eV for PnTX-G (**A**), SPX-1 (**B**), and GYM-A (**C**) and fragmentation pathways.

The LC-MS/MS method for lipophilic toxins described by Braña-Magdalena et al. 2014 [25] was modified for the specific analysis of CIs. A chromatography with a cycle time of 5 min was used for the separation of compounds. Under acidic mobile phase conditions, the imine group has a positive charge, allowing a rapid elution in reversed-phase chromatography. With the applied gradient, all the CIs in the study were baseline separated with an adequate resolution (supporting information, Figure S1). Mass spectrometry detection was performed in positive mode in which CIs are easily ionizable. The product ion spectra of [M + H]^+^ ions obtained for GYM-A, SPX-1 and PnTX-G are presented below (Figure 3). The results are in agreement with the fragmentation pathways proposed in the references consulted [28,29,30]. PnTXs and SPXs are closely related structural compounds [31]. In addition, it is supposed that PtTXs, like the rest of PnTXs, are produced from precursor PnTXs (F and G) by means of metabolic pathways in shellfish [32]. Therefore, SPXs, PnTXs, and PtTXs have similar structures and related *m*/*z* ions. The *m*/*z* 164 fragment ion (C_11_H_18_N^+^) is common to all reported pinnatoxins and pteriatoxins in addition to the majority of spirolides, and a *m*/*z* 572 fragment ion can be utilized as a PnTX- and PtTX-specific product ion for selected reaction monitoring (SRM) detection and confirmation of these toxins in samples [26,29]. In relation to GYMs, both the *m*/*z* 136 (C_9_H_14_N^+^) and the *m*/*z* 162 (C_11_H_16_N^+^), fragment ions with the CI moiety, can also be used for the screening of gymnodimine analogs [30,33].

### 3.2. Quality Control in LC-MS/MS Analysis

LC-MS/MS was evaluated using standard solutions prepared in MeOH (LC-MS grade) and matrix-matched standards (MMSs) prepared in a blank mussel extract without the application of the further SPE step. Data were obtained in dynamic multiple reaction monitoring (DMRM) that allowed for the acquisition of SRM data only within a selected retention time window, thereby improving both sensitivity and reproducibility. Limits of detection (LOD) and quantification (LOQ) were calculated based on an S/N > 3 and an S/N > 10, respectively, using triplicate injections (*n* = 3) of standard solutions with concentrations near the limits. The sensitivity reached in solvent was slightly higher than that obtained in MMSs, in which all the CIs were unambiguously detected at 0.3 µg/kg and quantified at 1 µg/kg. Linearity was evaluated by nine-point calibration curves prepared in both MeOH and MMSs within the range of 0.1 ng/mL to 40 ng/mL, obtaining a good adjusted linear regression (r^2^ ≥ 0.997). The matrix effect was also evaluated in this experiment, and a signal suppression was observed for all the CIs in the study, with the signal suppression being more intense in the case of PnTX-G (Table 2). Despite slight variations, the influence of matrix compounds did not affect significantly at ion ratios, which were reproducible. Retention time drift was evaluated between sets of calibration (*n* = 3), obtaining values <1%. SRM chromatograms of solvent blanks injected after calibration sets and between hydrolyzed extracts did not show signals due to carry over effects.

### 3.3. Quantification of SPX-1 and PnTX-G in Samples

Sample extracts from mussels (*Mytilus galloprovincialis*) harvested from three different locations of the Atlantic coast of Spain were analyzed, and SPX-1 and PnTX-G were detected in the eighteen available samples. The results obtained are summarized in Table 3. The concentrations took into account the matrix effect using MMSs prepared in a blank mussel extract for quantification. Samples from Ría de Sada (12 of 18) showed higher concentration levels for SPX-1, with a maximum of 6.9 µg/kg, while PnTX-G was present in higher amounts in samples from Ría de Arousa (6 of 18), with values in the range of 2.3–3.1 µg/kg. The variation of the concentration levels of PnTXs in relation to spatio-temporal changes in shellfish from the Atlantic and Cantabrian coasts of Spain was recently evaluated, and the maximum concentrations did not exceed 15 µg PnTXs/kg [19]. In addition, another recent work also reported the presence of PnTX-G in mussels from Galicia at levels similar to those found in this study [20].

In relation to the analysis of hydrolyzed extracts, the concentration of PnTX-G increased after alkaline hydrolysis within 3% to 22%, suggesting the presence of esterified forms of the toxin in samples. These results are in agreement with the ones obtained for the analysis of other mussels from this area, with the previous results showing that the concentration of free PnTX-G increased after hydrolysis but less than 30% [19].

### 3.4. LC-MS/MS Screening of CIs after the Concentration of Sample Extracts Using an SPE Step

Due to the low concentration levels of SPX-1 and PnTX-G found in the samples, with values close to the LOQ, a solid phase extraction (SPE) step was used for the concentration of extracts before the analysis of other CIs without available reference standard solutions (for supporting information, see Figure S2). The use of the SPE in this study was only for qualitative purposes, so an exhaustive recovery study was not carried out. Nevertheless, this step was slightly optimized and evaluated. With the SPE procedure used, load and wash fractions were analyzed without detection of loss of analytes in these steps. Recovery experiments were carried out using the sample extracts previously analyzed, obtaining recoveries for SPX-1 and PnTX-G of 75% and 89%, respectively. This preconcentration factor (i.e., 10 mL → 1 mL) provided the best recovery results, although an increase in concentration levels can be achieved by including a further evaporation step and a reconstitution in a volume of 0.5 mL, which slightly improved sensitivity. The recoveries obtained for CIs in these experiments were below 100%, most likely due to a matrix effect or the retention of CIs in the cartridge. After the concentration step, sample extracts were used in this study for the screening of CI analogs. The SPX group is composed of sixteen analogs tagged from A to I with some methylated, demethylated, and hydroxylated forms of SPX-C, SPX-D, and SPX-G [17]. The PnTX group consists of eight compounds named from A to H [34]. The PtTX group has three elucidated structures with the same molecular weight, PtTX-A in addition to the epimers B and C [35]. The GYM group has five analogs named from A to E, with some methylated forms of GYM-A [30,33,36]. GYMs, SPXs, PnTXs, and PtTXs, which did not have available reference standard solutions, were monitored based on fragmentation pathways and references reviewed, combining different modes in mass spectrometry analysis. The key ions previously identified were used for the MS analysis. The 164 *m*/*z* fragment ion was used in precursor ion mode within a scan range of 600–850 *m*/*z* for the screening of SPX, PnTX, and PtTX analogs, as well as a product ion in SRM analysis. In these experiments, PnTX-A was detected in samples from Ría de Arousa, while this compound was not detected in samples from Ría de Sada, most likely due to the low levels of PnTX-G in these samples. Based on proposed fragmentation pathways, three SRM transitions were selected for the detection of PnTX-A in mussel sample extracts (712 → 164; 712 → 458; 712 → 572) (for supporting information, see Figures S3 and S4). Other GYMs, SPXs, PnTXs, or PtTXs were not detected in the analyzed samples.

## 4. Conclusions

The application of LC-MS/MS for the analysis of CIs allowed for the identification of SPXs and PnTXs in mussels from Galician Rias, Spain. The results obtained in the quality control performed with certified reference standard solutions of GYM-A, SPX-1, and PnTX-G were satisfactory, indicating that, after carrying out an extension of the validation of the reference method, these compounds could be regularly monitored by LC-MS/MS as well as other groups of lipophilic toxins currently legislated. The EURLMB SOP for lipophilic toxins was used for the confirmation and quantification of SPX-1, PnTX-G, and the indirect determination of esterified forms of PnTX-G. Finally, an SPE step was included in the sample treatment in order to concentrate the extracts before screening for analogs of CIs. Additionally, other GYMs, SPXs, PnTXs, and PtTXs without available standard solutions were analyzed based on fragmentation pathways and key ions, using different modes of data acquisition in mass spectrometry analysis, and identifying the presence of PnTX-A in the samples with higher levels of PnTX-G. These results provide more data about the application of LC-MS/MS for determining the occurrence of CIs in shellfish from Galician Rias, Spain.

## Figures and Tables

**Table 1:** MS/MS conditions used for the dynamic multiple reaction monitoring (DMRM) acquisition of data for cyclic imines (CIs) on a 6460 Agilent Technologies mass spectrometer with a retention window of 0.5 min.

Analyte	Precursor Ion, *m*/*z*	Product Ion, *m*/*z*	Fragmentor, V	Collision Energy, V	Cell Accelerator	Retention Time, min
GYM-A	508.3	392.3	220	40	3	2.60
GYM-A	508.3	136.1	220	42	3	2.60
desmethylSPX-1	678.5	430.3	240	40	2	3.11
desmethylSPX-1	678.5	164.1	240	54	2	3.11
SPX-1	692.5	444.3	240	40	2	3.68
SPX-1	692.5	164.1	240	54	2	3.68
PnTX-A	712.4	458.3	240	50	2	2.87
PnTX-A	712.4	164.1	240	60	2	2.87
PnTX-G	694.5	458.3	240	50	2	4.55
PnTX-G	694.5	164.1	240	60	2	4.55

**Table 2:** Mean slope and relative standard deviation (RSD) for a nine-point calibration curve for CIs using methanol and matrix-matched standards (MMSs); calibration curve data is the mean of three sets of calibration ranging from 0.1 to 40 ng/mL.

	Methanol		MMS		
Analyte	Slope	RSD, % *	Slope	RSD, % *	Signal Suppression, %
PnTX-G	135,853	2.2	98,659	2.9	27.4
SPX-1	226,904	0.8	225,225	2.6	0.7
GYM-A	40,854	1.4	36,309	2.6	11.1

* *n* = 3.

**Table 3:** Concentration of CIs (μg/kg) obtained in the LC-MS/MS analysis of samples following European Union Reference Laboratory for Marine Biotoxins (EURLMB) standard operating procedure. LoQ: limit of quantification.

Sampling Zone	Sample Code	SPX-1 (µg/kg)	PnTX-G (µg/kg)	
			Before Hydrolysis	After Hydrolysis
Ría de Sada A (1)	RS1.1	2.7	1.8	2.2
	RS1.2	1.9	1.8	2.2
	RS1.3	3.1	1.8	2.2
	RS1.4	2.2	1.8	2.2
	RS1.5	3.3	1.8	2.2
	RS1.6	3.5	1.8	2.2
	RS1.7	4.0	1.8	2.2
	RS1.8	1.8	1.8	2.2
	RS1.9	6.9	1.8	2.2
	RS1.10	4.2	1.8	2.2
	RS1.11	4.2	1.8	2.2
	RS1.12	3.5	1.8	2.2
Ría de Arousa (2)	RA2.1	<LoQ	2.9	3.2
	RA2.2	<LoQ	3.1	3.2
Ría de Arousa (3)	RA3.1	1.2	2.3	2.7
	RA3.2	1.6	2.8	3.1
	RA3.3	<LoQ	2.3	2.7
	RA3.4	2.2	2.9	3.2

## Supplementary Materials

The following are available online at https://www.mdpi.com/1660-4601/17/1/281/s1: Figure S1: Representative dynamic multiple reaction monitoring (DMRM) chromatogram of CIs; Figure S2: Overlapped total ion chromatogram (TIC) of sample RA3.4 before and after SPE; Figure S3: Structure of PnTX-A and fragment ions selected for multiple reaction monitoring (MRM) analysis; Figure S4: Multiple reaction monitoring (MRM) chromatograms for PnTX-A acquired on sample RA3.4 after SPE.
