# Supplementary material for: Analysis of Cyclic Imines in Mussels (Mytilus galloprovincialis) from Galicia (NW Spain) by LC-MS/MS
Source: Int J Environ Res Public Health. 2019 Dec 31;17(1):281. doi: 10.3390/ijerph17010281 (PMC6981759; doi:10.3390/ijerph17010281)
Supplement: Supplementary file 1 [file ijerph-17-00281-s001.pdf]

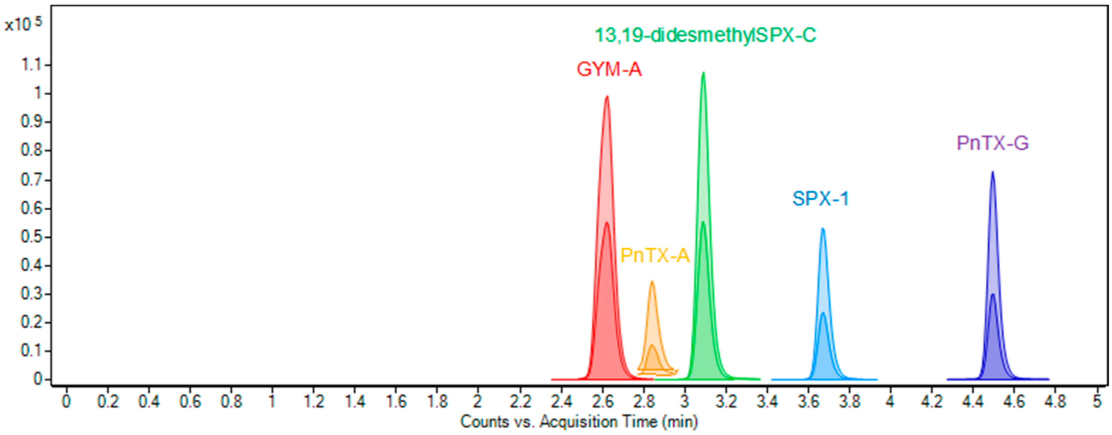

Figure S1. Representative dynamic multiple reaction monitoring (DMRM) chromatogram of CIs.

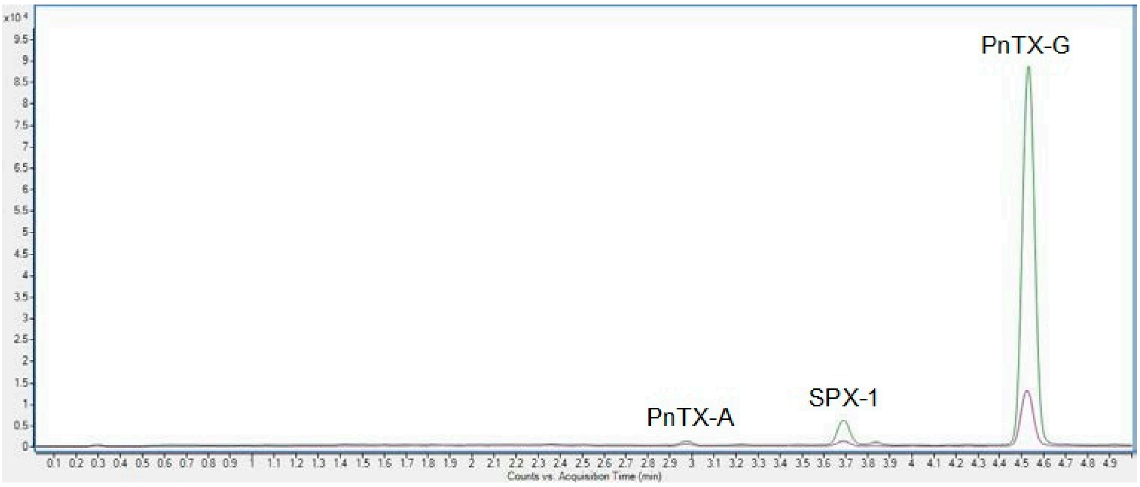

Figure S2. Overlapped total ion chromatogram (TIC) of sample RA3.4 before and after SPE.

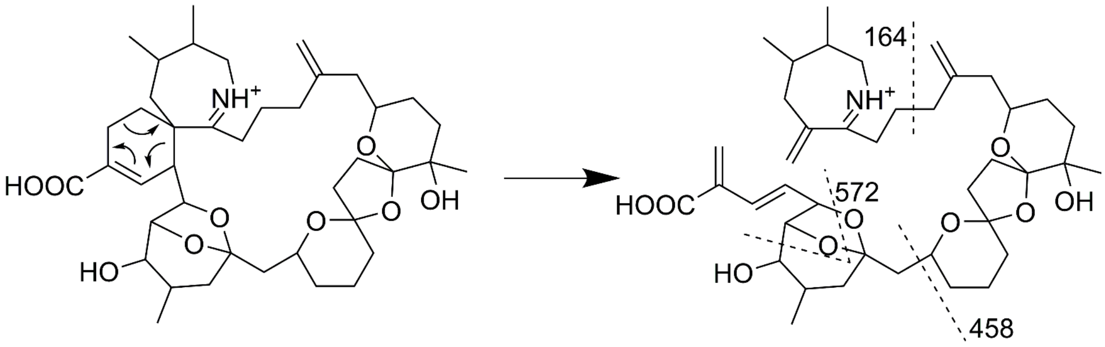

Figure S3. Structure of PnTX-A and fragment ions selected for multiple reaction monitoring (MRM) analysis.

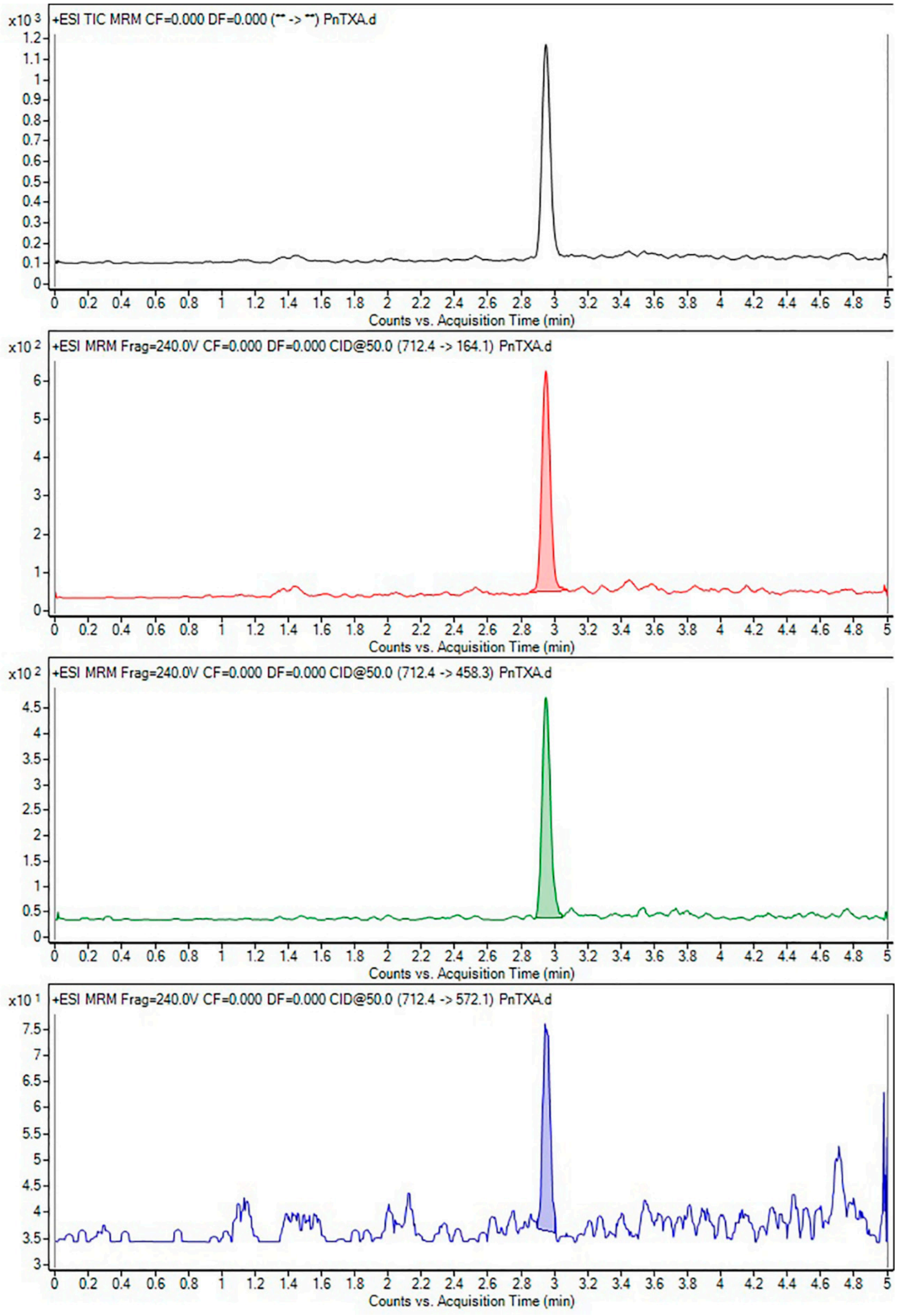

Figure S4. Multiple reaction monitoring (MRM) chromatograms for PnTX-A acquired on sample RA3.4 after SPE.
